# Young’s Modulus-Independent Determination of Fibre Parameters for Rayleigh-Based Optical Frequency Domain Reflectometry from Cryogenic Temperatures up to 353 K

**DOI:** 10.3390/s23104607

**Published:** 2023-05-09

**Authors:** Caroline Girmen, Clemens Dittmar, Thorsten Siedenburg, Markus Gastens, Michael Wlochal, Niels König, Kai-Uwe Schröder, Stefan Schael, Robert H. Schmitt

**Affiliations:** 1 Department Production Metrology, Fraunhofer Institute for Production Technology IPT, Steinbachstraße 17, 52074 Aachen, Germany; 2 I Physics Institute B, RWTH Aachen University, Templergraben 55, 52062 Aachen, Germany; 3 Institute of Structural Mechanics and Lightweight Design, RWTH Aachen University, Templergraben 55, 52062 Aachen, Germany

**Keywords:** optical fibre sensor, OFDR, calibration, strain, cryogenic temperature, AMS-100

## Abstract

The magnetic spectrometer AMS-100, which includes a superconducting coil, is designed to measure cosmic rays and detect cosmic antimatter in space. This extreme environment requires a suitable sensing solution to monitor critical changes in the structure such as the beginning of a quench in the superconducting coil. Rayleigh-scattering-based distributed optical fibre sensors (DOFS) fulfil the high requirements for these extreme conditions but require precise calibration of the temperature and strain coefficients of the optical fibre. Therefore, the fibre-dependent strain and temperature coefficients $K_{T}$ and $K_{\epsilon }$ for the temperature range from 77 K to 353 K were investigated in this study. The fibre was integrated into an aluminium tensile test sample with well-calibrated strain gauges to determine the fibre’s $K_{\epsilon }$ independently of its Young’s modulus. Simulations were used to validate that the strain caused by changes in temperature or mechanical conditions was the same in the optical fibre as in the aluminium test sample. The results indicated a linear temperature dependence of $K_{\epsilon }$ and a non-linear temperature dependence of $K_{T}$. With the parameters presented in this work, it was possible to accurately determine the strain or temperature of an aluminium structure over the entire temperature range from 77 K to 353 K using the DOFS.

## 1. Introduction

The demand for optical fibre sensors has increased in recent years due to their unique capabilities and advantages over conventional sensors [1]. One of the main reasons for this is their high sensitivity, which enables them to detect small changes in physical parameters such as temperature, pressure, strain, and displacement [2]. In addition, fibre optic sensors are immune to electromagnetic interferences, so they can be used in environments where conventional sensors, such as strain gauges or temperature resistors, would be affected [3]. Another reason for the increasing demand for fibre optic sensors is their versatility and wide range of applications in various industries. They are used in telecommunications to monitor the performance of optical networks, in the oil and gas industry to monitor the integrity of pipelines [4], in aerospace to monitor the structural conditions of aircraft [5], and in civil engineering to monitor the deformation of bridges and other structures [6].

In recent years, this wide range of applications has been increasingly extended to space applications, where the reliable monitoring of critical structural elements is essential [7]. Especially for extreme environments such as cryogenic temperatures, optical fibres are an important technology for structural health monitoring [8,9].

Overall, fibre optic sensors offer a combination of high accuracy, immunity to electromagnetic interferences, lightweight and compact design, durability, multi-parameter sensing, and a long range that make them ideal to use for the harsh environment in space applications, such as for use with the magnetic spectrometer AMS-100 [10].

Fibre optic sensors use the properties of light to measure various physical parameters. Two common types of fibre optic sensors are FBGs (fibre Bragg gratings) [11] and distributed sensing, which can be based on Brillouin, Raman, or Rayleigh scattering [12,13], while FBGs comprise small, periodic variations in the refractive index of the optical fibre that are inscribed in the fibre core and are able to perform multiple point measurements. Rayleigh-based distributed sensing uses the inherent scattering of light within the optical fibre, known as Rayleigh scattering, to measure changes in the physical parameters along the entire length of the fibre. The backscattered light is collected and analyzed to determine the location and magnitude of the changes [14]. Rayleigh-based distributed sensing is less sensitive than FBGs, but it can measure changes along the entire length of the fibre. Sensitivity to multiple parameters is both an advantage and a disadvantage. Due to the coupling of temperature and strain, no simultaneous determination of the individual parameters can be guaranteed if both occur in parallel. To decouple both parameters, different methods can be used, such as the mechanical decoupling of a second fibre [15], the use of a single distributed optical fibre ring [16], or mathematical decoupling with precise calibration [7]. This calibration requires exact knowledge of the fibre-dependent temperature and strain parameters. For both FBGs and Rayleigh-based sensors, the cross-sensitivity is caused by an additional thermal signal due to the thermo-optical effect. The strength of the purely thermal signal depends on the doping of the light guide.

This work will cover the determination of the fibre-dependent strain and temperature coefficient for distributed Rayleigh-based OFDR measurements. The advantage of the proposed method in comparison to studies of the past [17,18] is that the new method can be used for distributed decoupled temperature and strain measurements in cryogenic temperatures instead of single-point FBG measurements. In previous work [19], the fibre parameters for distributed sensing were determined through a low-temperature test device based on mechanical loading through weights. The drawback of this method was the dependency on the exact knowledge of the Young’s modulus of the optical fibreglass. To overcome these issues, a cryogenic tensile testbed was built to measure the applied strain through the spectral shift and additional applied strain gauges.

The proposed method for measuring distributed temperatures for cryogenic temperatures in the literature [19] and in this study is based on Rayleigh scattering, which occurs in single-mode optical fibres [13]. When light strikes an inhomogeneous medium whose dimensions are smaller than the wavelength of the light, Rayleigh scattering occurs. In the case of an optical fibre, random variations in the refractive index of the fibre material (glass) cause Rayleigh scattering. When the temperature changes, the refractive index and the length of the optical fibre also change, thereby causing shifts in the Rayleigh backscatter spectra. In addition to that applied strain to the fibre, temperature changes also result in a change in the optical path of the backscattered light and cause a measurable shift in the spectrum.

(1) $$-\;\frac{\Delta \nu }{\hat{\nu }}\;=\;K_{T}\cdot \Delta T\;+\;K_{\epsilon }\cdot \epsilon$$

Equation (1) describes the basic model for spectral shift decoupling in temperature change and strain. $K_{T}/K$ is the temperature sensitivity, and $K_{\epsilon }$ is the strain sensitivity of the optical fibre to longitudinal strain applied to the sensor fibre. It is the linear description of the fibre signal for thermal and mechanical load. The approximate linear behaviour can be derived from the spectral shift of a Bragg grating with variations of 10% [20]. The measured spectral shift Δν is the frequency change of the measured signal for a fibre under test compared to the reference measurement, while $\hat{\nu }$ is the central frequency of the tunable laser sweep range frequency of a OBR-4613 instrument [14]. ΔT is the thermal load as the difference of the measurement temperature to the reference temperature, and ϵ is the strain as the normalised length change between the reference and measurement.

The increasing range of application areas for optical fibre sensors results in the need for more precise modelling and the separation of the actual temperature and strain applied to the fibre. Therefore, the fibre constants need to be determined precisely and calculated with a more complex model, which will be discussed in more detail in the next sections.

This paper was written in parallel to a master’s thesis by Clemens Dittmar [21]. The theoretical derivation of the thermal and mechanical decoupling, the description of the experimental setups, the analysis of the data, and the description of the results, as well of the fibre optic measurements, are extracts from the master’s thesis.

## 2. Principle and Experimental Setup

The setup includes an OBR-4613 from Luna Technology, which is a Keithly 2701 multimeter for read-out of strain gauges, thermal resistors (Pt1000), and thermally insulated chambers for slow warm-up and cryogenic tensile testing.

### 2.1. Non-Linear Calculation Model for Thermal and Mechanical Loading Effects in OFDR Signals

As mentioned in the introduction, the basic model for calculating strain and temperature in Rayleigh-based OFDR measurements does not cover more complex use cases. Therefore, a more complex, non-linear model is needed. The standard model is a linear approximation of Equation (2) for weak FBGs.

(2) $$-\;\frac{\Delta \nu }{\hat{\nu }}\;=\;\int_{T_{R}}^{T_{M}}K_{T}\left(T\right)\;dT\;+\;K_{\epsilon }\cdot \epsilon$$

$K_{T}$ (Equation (3)) for a free fibre is generally the sum of the coefficient of thermal expansion of the whole fibre, $\alpha_{Fibre}$ and the thermal optical coefficient (TOC), the normalised thermal dependence of the refractive index [22], of the core.

(3) $$K_{T}\left(T\right)\;=\;\alpha_{Fibre}\left(T\right)\;+\;\frac{1}{n}\frac{dn}{dT}$$

As Wang et al., explained by applying Gosh’s theory for the temperature dependence of the refractive index for glasses, the TOC of silica has a temperature dependency that is related to the band gap energy structure [23]. They showed the complex relationship as visible in Equation (4), which can be approximated by a polynomial of at least 1 order or higher [22].

(4) $$K_{T}\left(T\right)\;=\;\alpha_{Fibre}\left(T\right)\;+\;\frac{1}{n}\frac{dn}{dT}\left(T\right)\;\approx \;\alpha_{Fibre}\left(T\right)\;+\;A\;+B\cdot T\;+\;O\left(T^{2}\right)$$

For a free fibre, the coefficient of thermal expansion (CTE, $\alpha_{\mathrm{Fibre}}$) of the whole fibre can be considered as part of the thermal sensitivity. However, if the fibre is bonded to a sufficiently stiff substrate, the CTE of the substrate is imposed on the fibre, thereby changing the thermal sensitivity if the CTE is still part of it. Therefore, in the following, the thermal sensitivity was defined by the TOC only (Equation (5)), and a thermo-mechanical mixing term $K_{M}$ was obtained (Equation (6)).

(5) $$K_{T}\left(T\right)\;=\;\frac{1}{n}\frac{dn}{dT}\left(T\right)\;\approx \;A\;+B\cdot T\;+\;O\left(T^{2}\right)$$

$K_{M}$ is a product of the substrate CTE and $K_{\epsilon }$, since the thermally induced mechanical signal can be considered as an external mechanical load acting on the fibre.

(6) $$K_{M}\left(T\right)\;=\;K_{\epsilon }\left(T\right)\cdot \alpha_{Sub.}\left(T\right)$$

$K_{\epsilon }$ has a temperature dependence, which, according to Lu et al. [24], can be parameterised by Equation (7).

(7) $$K_{\epsilon }\left(T\right)\;=\;1\;-\;\frac{n{\left(T\right)}^{2}}{2}\cdot \left(P_{12}\left(T\right)\;-\;\mu \left(T\right)\cdot \left(P_{11}\left(T\right)\;+\;P_{12}\left(T\right)\right)\right)$$

where $P_{11}$ and $P_{12}$ are the Pockel coefficients of the stress-optical tensor, and μ(T) is the Poisson ratio of the fibre. It is important to note that all elements of Equation (7) have a temperature dependence and strain sensitivity [25]. This was theoretically determined for FBGs by Martin et al. [25] for the range 400 K–700 K and measured for differently doped FBGs. It was found that the temperature dependence could be approximated linearly, and the slope was, on average, $\left(2.1\;\pm \;0.03\right)\;\cdot \;10^{-4}$/K. Linear temperature dependence of $K_{\epsilon }$ was, therefore, assumed in the following Equation (8).

(8) $$K_{\epsilon }\left(T\right)\;=\;m\cdot T\;+\;b$$

Together with the previously determined temperature dependency of $K_{T}$, a general decoupling Equation (9) for the spectral shift is given.

(9) $$\begin{array}{cc} -\;\frac{\Delta \nu }{\hat{\nu }}\; & =\;\int_{T_{R}}^{T_{M}}K_{T}\left(T\right)\;dT\;+\;\int_{T_{R}}^{T_{M}}K_{M}\left(T\right)\;dT\;+\;K_{\epsilon }\left(T_{M}\right)\cdot \epsilon_{External}\left(T_{M}\right)\\ -\;\frac{\Delta \nu }{\hat{\nu }}\; & =\;F\left(T_{M},T_{R}\right)\;+\;G\left(T_{M},T_{R}\right)\;+\;K_{\epsilon }\left(T_{M}\right)\cdot \epsilon_{External}\left(T_{M}\right) \end{array}$$

$\epsilon_{External}\left(T_{M}\right)$ is a non-thermal, mechanical strain from an external source at measurement temperature $T_{M}$. The thermal signal function is given as $F(T_{M},T_{R})$. and $G(T_{M},T_{R})$ is the thermo-mechanical signal function used in the following data analysis. $F(T_{M},T_{R})$ is given in Equation (10), which includes non-linearities up to second order.

(10) $$F\left(T_{M},T_{R}\right)\;=\;\int_{T_{R}}^{T_{M}}K_{T}\left(T\right)\;dT\;=\;A\cdot \left(T_{M}\;-\;T_{R}\right)\;+\;\frac{B}{2}\cdot \left(T_{M}^{2}\;-\;T_{R}^{2}\right)\;+\;O\left(T^{3}\right)$$

For a linear approximation of $K_{\epsilon }\left(T\right)$, the integral $G(T_{M},T_{R})$ can be simplified with the mean value of $T_{M}$ and $T_{R}$.

(11) $$\begin{array}{cc} G(T_{M},T_{R})\; & =\;\int_{T_{R}}^{T_{M}}K_{\epsilon }\left(T\right)\cdot \alpha_{Sub.}\left(T\right)\;dT\;\\ \; & \approx \;K_{\epsilon }\left(\frac{T_{M}\;+\;T_{R}}{2}\right)\cdot \int_{T_{R}}^{T_{M}}\alpha_{Sup.}\left(T\right)\;dT\\ \; & =\;K_{\epsilon }\left(\frac{T_{M}\;+\;T_{R}}{2}\right)\cdot \epsilon_{Sub.}\left(T_{M},T_{R}\right) \end{array}$$

For small temperature changes, an approximation with constant temperature is, therefore possible. $\epsilon_{Sub}\left(T_{M},T_{R}\right)$ is the thermo-mechanical strain of the substrate. Finally, Equation (12) approximates an optical fibre bonded to a substrate with all thermal dependencies taken into account.

(12) $$\begin{array}{cc} -\;\frac{\Delta \nu }{\hat{\nu }}\;= & \;A\cdot \left(T_{M}\;-\;T_{R}\right)\;+\;\frac{B}{2}\cdot \left(T_{M}^{2}\;-\;T_{R}^{2}\right)\;+\;O\left(T^{3}\right)\\ \; & +\;\left(m\cdot \frac{T_{M}\;+\;T_{R}}{2}\;+\;b\right)\cdot \epsilon_{Sub.}\left(T_{M},T_{R}\right)\\ \; & +\;\left(m\cdot T_{M}\;+\;b\right)\cdot \epsilon_{External}\left(T_{M}\right) \end{array}$$

It should be noted that this equation covers thermo-mechanical signals, mechanical signals at constant temperature, and thermal signals at constant strain, but it does not cover external mechanical signals at varying temperatures. However, if the additional temperature change is small, this equation is a sufficient approximation for these cases as well, since the additional thermal expansion is considered.

### 2.2. Optical Frequency Domain Reflectometry Interrogator

**Figure 1:**
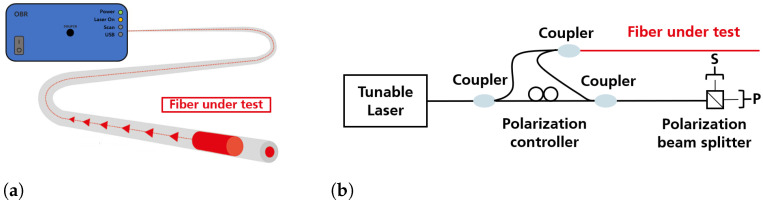
Schematic (**a**) image of the measurement principle of the LUNA OBR device and (**b**) simplified setup of the Luna OBR measurement device. (Adapted from [27,28]).

The Luna OBR 4613 was used to measure the Rayleigh spectral shift. It has one channel with a fibre range of 70 m (limited by the software), a spatial resolution of 0.6 mm, and a sensitivity of 1 microstrain (μm/m) and 1 °C. The Luna OBR system was chosen as the interrogation system for the determination of the OFDR-relevant fibre parameters due to its high resolution and flexibility to perform measurements in various locations, as it has a portable design. In continuous measurement mode, the software automatically performs OFDR measurements at predefined intervals. The shortest interval used here was one second. A Python script was written for which a measurement period and a fixed number of measurements could be defined. A tunable laser source, most simply with a ramp waveform, emitted light that was coupled into an optical fibre. The laser source in the LUNA OBR 4613 emits 10 mW in a possible tuning range of 1260 nm to 1340 nm. The intensity was split into two paths by a coupler that allows a light beam to be split into two paths. One path led to the interferometric measurement setup shown in Figure 1, which is sufficient for performing OFDR measurements. In the upper measurement arm, the light was coupled into the fibre under test (FUT), which acted as the actual sensor. In the FUT, the light propagates through the entire fibre. Rayleigh scattering events along the entire length of the FUT cause photons to be emitted in all directions. Some of these may travel back through the fibre to the coupler by total internal reflection [26].

The polarisation controller provided equal intensities for the two polarisation axes coming from the reference arm. The light returning from the FUT had arbitrary polarisation states and was coupled with the light from the reference arm. The light was split into two perpendicular polarisation states by a polarising beam splitter (PBS), and the intensity of the interfering signals was measured by photodiodes connected to a data acquisition board. This ensured that the interference between the light from the measurement and the reference arm was measured as a vector sum of the obtained signal in both detectors. When the laser wavelength is tuned, a periodic signal is generated at the detector, and its frequency depends on the position of the respective backscattering fibre segment. Backscattered light from positions further away along the fibre corresponds to higher frequencies. The signal sum of all segments along the fibre was decomposed into its frequency components by Fourier transformation. The frequencies then correspond to the signal locations in the fibre. The amplitude of each frequency component indicates the strength of the respective reflection [27,28].

### 2.3. Strain Gauge Sensor

The measurement of thermal and mechanical strain was carried out using strain gauges from Hottinger Brüel and Kjaer GmbH (HBM), which consist of a 350 Ω resistance grid that is sensitive to mechanical and thermal influences. In order to compensate for temperature-dependent resistance changes when the strain gauge is applied to steel, the manufacturer provides a compensation curve for each strain gauge size. Therefore, a strain gauge must always be read with a temperature sensor. The grid has a CTE equal to that of steel, and the sensor signal must be compensated if a substrate other than steel is used, as the temperature correction assumes steel as the sensor target. To determine a correction function, the strain gauges were calibrated on two materials with well-known thermal strain characterisations (NIST data). Titanium (Ti-6Al-4V) [29] was used for the temperature range 77 K–290 K, while pure silicon [30] from a spare AMS-02 silicon wafer was used for 290 K–353 K. Thermal strain characterisations for both materials are available from the NIST standard reference database for their respective temperature ranges. Silicon, however, could not be used for the low temperature range, because its grain boundaries could not withstand the thermal stresses caused by the strain gauge, thereby resulting in delamination along with a silicon layer. Titanium could not be used in the range above 300 K, because the NIST data only goes up to 300 K. Calibration was performed with slow warm-up measurements from 77 K to 300 K and measurements from 233 K to 353 K in a climate chamber (Binder MK240/CTS 70-1500).

Class A Pt1000 platinum resistors were used for temperature measurement. These were read out using the four-wire technique with a Keithly 2701 multimeter. Resistance was converted to temperature with the Callendar van Dusen Equation [31]. The statistical uncertainty of the temperature measured by the Pt1000 was 0.01 K, and the systematic uncertainty was 0.15 K + 0.6%·|TK − 273.15|. The statistical uncertainty on the strain measurements was dominated by the statistical temperature uncertainty, which was included in the calculation of the strain values from the resistance data.

To test the strain gauge calibration, the thermal strains of Aluminium-6061-T6511 and oxygen-free copper were measured, as their thermomechanical behaviour is known from the NIST database [32,33]. The measurements were carried out for each sample with a strain gauge and a Pt1000 using the same procedure as for the calibration.

**Figure 2:**
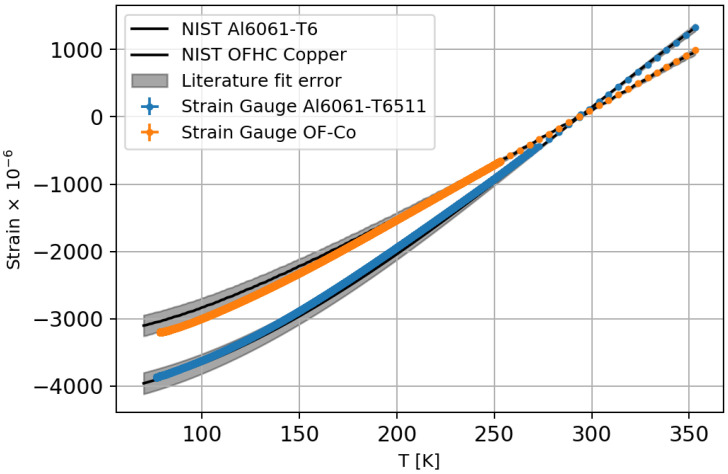
The first plot shows the strain gauge measurement of Al-6061-T6511 and OF-Copper, as well as the NIST characterisations for these materials.

The results of the measurements are shown in Figure 2, together with the corresponding NIST characterisation. The calibrated strain gauges were used to determine the thermo-mechanical strain of the Aluminium 6060 fibre carrier material. The parameters used in this work are listed in Section 3.1. The systematic uncertainty on the strain measured by the strain gauge sensor and on the thermo-mechanical strain for the Aluminium 6060 parametrisation, measured with strain gauge sensors, was estimated with $4\times 10^{-6}\;+\;0.6\%\times \mathrm{Strain}$.

### 2.4. Cryogenic Tensile Tests

**Figure 3:**
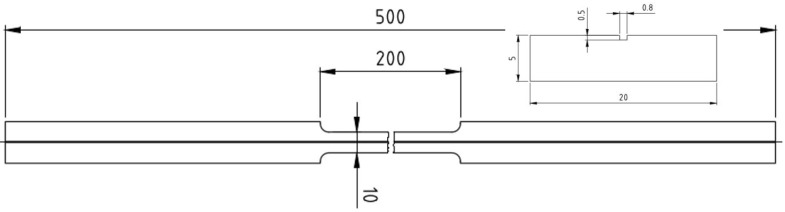
Technical drawing of the manufactured test sample made of aluminium with milled groove into which the fibre is be glued.

For the measurements, a test sample was manufactured from Aluminium 6060 with the geometry shown in Figure 3.

The fibre used here is a SM1500(9/125)P germanium-doped single-mode fibre with core, cladding, and coating dimensions of (9/125/157 μm) with a polyimide coating terminated with a pigtail. It was glued into a groove parallel to the longitudinal axis of an aluminium tensile specimen with an epoxy adhesive (Scotch–Weld 2216 grey) [34]. The adhesive and the coating where chosen because of their resistance against huge temperature changes and cryogenic temperatures [35,36]. According to the manufacturer’s data sheet [34] and our experience with the Scotch–Weld 2216 grey adhesive, it can transfer the mechanical behaviour of the substrate to the fibre, even between 77 K–353 K. The specimens were tested on an Instron 5567 electrical tensile testing machine with a 30 kN load cell. Wedge grips were used for specimen gripping, which automatically tighten under tensile load and counteract the loss of gripping force due to the thermal expansion of the specimen. The central, tapered area was the measuring area, and the specimen was clamped in the tensile machine at the two wider ends. The strain was measured with a strain gauge from HBM, which was placed centrally in the middle area. Another strain gauge was placed centrally but orthogonal to the longitudinal axis of the specimen. Additional Pt1000 temperature sensors were placed next to the strain gauges.

**Figure 4:**
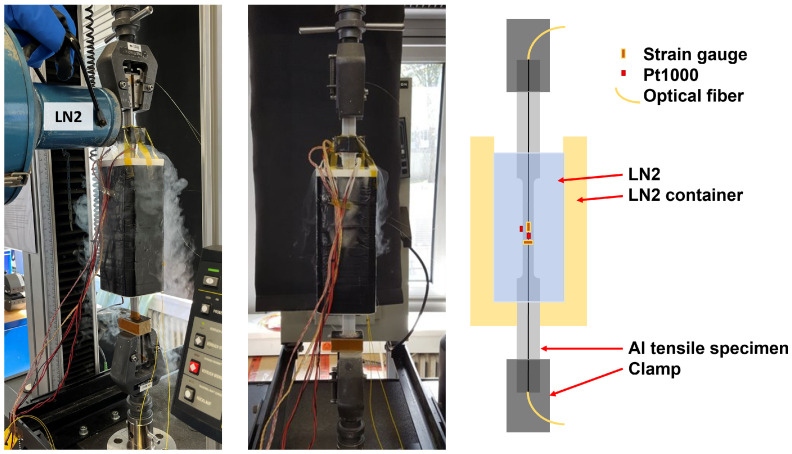
Tensile test with an aluminium specimen in an LN2 pox with temperature and strain sensors.

A liquid nitrogen container was placed around the central area of the strain gauge, enclosing the central 30.0 cm of the specimen. This allowed the strain specimen to be clamped into the tensile test machine and the central area to be cooled with liquid nitrogen. Figure 4 shows the tensile specimen and the tensile test setup. The tensile test machine has an integrated distance meter and load cell (30 kN), which was read out together with the Pt1000 and strain gauges with a Keithley 2701 multimeter with a measurement range of 120 MΩ and a resolution of 1 mΩ.

One series of measurements was carried out at room temperature and two at 77 K under load control. The tension was increased to 400 N steps over 30 s and held for 10 s. The maximum force was 3600 N.

### 2.5. Warm Up Chamber

For the warm-up experiment from 77 to 300 K, the aluminium tensile specimen was equipped with two Pt1000 temperature sensors at each of three positions for this test. These were thermally bonded to the aluminium at the top and bottom using copper tape and thermal paste. The OBR measurement system was controlled externally from a laboratory PC using a Python program to record one measurement per minute. The sample was placed inside a thermal box, which contained a steel-grid base with a large thermal capacity at the bottom and was supported by polycarbonate blocks to provide thermal insulation.

**Figure 5:**
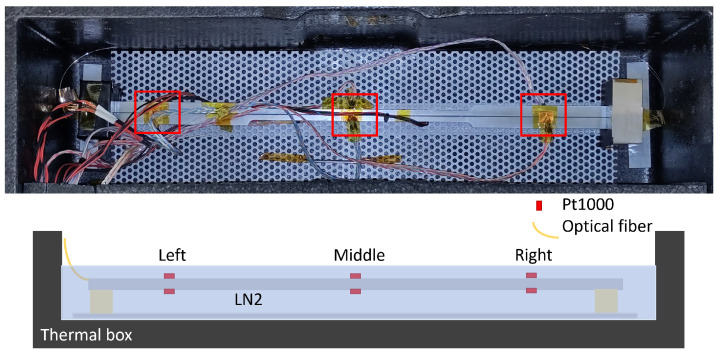
Aluminium specimen with 6 Pt1000 sensors—3 at the top and 3 at the bottom, with a germanium-doped fibre glued into the sample. The sample was placed in a thermal box with additional steel elements to slow down the heating process. The sketch shows a side view of the setup. The red boxes mark the positions of the Pt1000.

Figure 5 shows the setup for the warm-up measurements inside the thermal box. It was filled with liquid nitrogen and, once the temperature had stabilised at 77 K, the box was sealed with a plastic lid. The nitrogen gas produced by evaporation in the box was able to vent through smaller openings. The measurement took 18 h to reach room temperature.

For the higher temperature range, the specimen was installed in a climate chamber, and ten measurements were taken at each temperature level.

The climatic chamber was set to 6 different temperatures from 233 K to 353 K and held constant for 30 min until the Pt1000 data had settled.

### 2.6. Simulation

**Figure 6:**
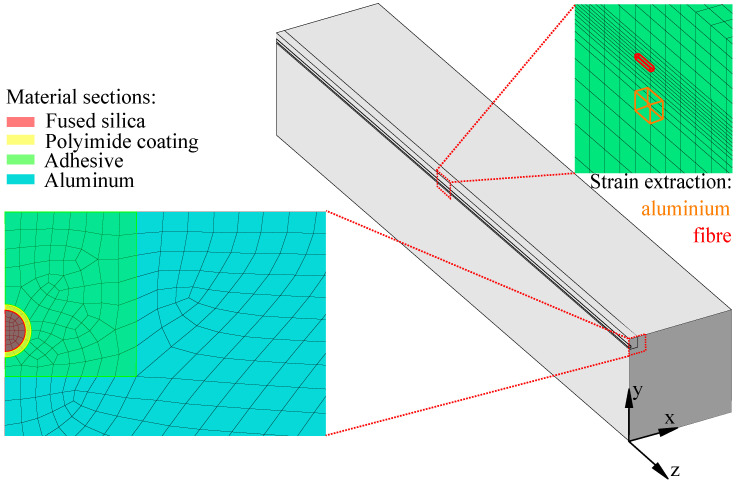
Detailed quarter simulation of fibre in aluminium with highlighted material sections.

To verify that the strain of the aluminium was well transferred to the fibre, a detailed 3D FEM simulation was performed in Abaqus 2020. In the simulation, the fibre (fused silica), the polyimide coating, the epoxy adhesive EP-50-5150, and the Aluminium 6060 were resolved in detail (see Figure 6) by C3D8R linear brick elements with reduced integration. The specimen was subjected to a temperature profile of a warm-up from LN2 to room temperature in several steps (77 K, 100 K, 200 K, and 300 K) with reference at 294.95 K. The procedure was derived from the warm-up tests described in Section 2.5.

A 3D model was chosen because, with the strain in fibre strain, the measured value is orthogonal to the cross-section. To reduce the computational effort, the axisymmetric specimen was simulated in a quarter model, which had a fixed point constraint at the origin of the coordinate system and symmetry boundary conditions in the x–y plane and y–z plane. The mirroring in the x–y plane by the chosen boundary conditions guarantees that the specimen can contract and expand unhindered under the influence of temperature, and, thus, no thermally induced stresses are applied by the constraints.

The mechanical material properties of aluminium [37], epoxy [35], and polyimide [36,38] used for the simulation can be found in Table 1. Temperature-dependent coefficent of thermal expansion curves were implemented to model realistic cooling and warm-up behaviour. For this purpose, strain measurements were taken on various materials during the cooling and warm-up processes on material specimens. The temperature–strain curves were fitted using a polynomial of the form

(13) $$\varepsilon \left(T\right)=B_{0}+B_{1}T+B_{2}T^{2}+B_{3}T^{3}+B_{4}T^{4},$$

and the fitting parameters $B_{0}$ to $B_{4}$ were extracted with an uncertainty of $4\times 10^{-6}\;+\;0.6\%\times \mathrm{Strain}$ for Aluminum 6060. These can be found in Table 2 and were converted for use in Abaqus.

For the simulation of the fibre, the literature values of fused silica were used. The thermal expansion coefficient of $2.7\times 10^{-7}$/K was taken from the literature, but it is only given for a temperature range of 223.15–273.15 K [38]. Literature data on the coefficient of thermal expansion down to 77 K were not found.

## 3. Results

The following section covers the results of the simulation, strain, and temperature sensitivity of the optical fibre.

### 3.1. Simulation

With the simulation of the linear sample described in Section 2.6, it could be shown that the fibre strain was comparable to the strain in the aluminium sample, with a deviation of 0.7% at 77 K and 2% after warm-up to 300 K. The simulated strain values in the z direction are given in Table 3 for the different temperature steps and are extracted from the middle of the specimen in the fibre’s centre and in the aluminium directly under the adhesive (Figure 6).

### 3.2. Strain Sensitivity

To determine the strain sensitivity, the mean value over 2 cm of the spectral shift at the position of the strain gauge was used and plotted against the measured strain from the strain gauge. Only data from 400 N upwards (after the first force step) were considered to avoid possible systematic effects caused by the self fixing of the clamps from the tensile measurement stand. The maximum temperature variation was 0.1 K at room temperature and 0.04 K and 0.05 K at 77 K.

**Figure 7:**
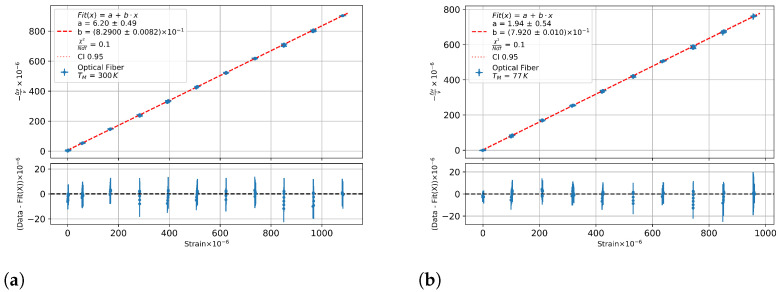
The spectral shift from an germanium-doped fibre against the measured strain (**a**) at room temperature and (**b**) at 77 K with increased maximum tension.

Figure 7a shows room temperature data, and Figure 7b shows a 77 K measurement and the slopes, i.e., the $K_{\epsilon }$ values for a specific temperature are listed in Table 4.

A linear fit to the strain sensitivities was performed, and the parameters are listed in Table 5.

### 3.3. Thermal Sensitivity

The OBR data from the warm-up measurement were processed with a reference at liquid nitrogen just before the liquid nitrogen level in the thermal box fell below the sample. This was 170 min after first LN2 contact, and only data from this data set onwards were used. The OBR data from the climate chamber measurement were processed with a reference at 293 K. The reference and measurement data sets were compared stepwise for the evaluation of the spectral shift. The step size was defined as the gauge length and gave the width of the data block used for the cross-correlation of spectral shift and time shift. The gauge length used was 2 cm with a resolution of 0.6 mm. An interquartile range filter (IQR) was applied to the fibre data to filter out the discontinuous peaks at higher temperature differences.

Equation (14) was used to calculate the thermal fibre signal, which was corrected for the thermo-mechanical component. The temperature data were used to calculate the thermo-mechanical strain, $\epsilon (T_{M},T_{R})$, with the thermal expansion of Al6060, which can be seen in Table 2. The temperature $T_{M}$ was calculated from the average of the upper and lower Pt1000 sensors. The error in the temperature was assumed to be the full difference between the upper and lower Pt1000 sensors. $T_{R}$ is the temperature of the reference OBR data set.

(14) $$-{\frac{\Delta \nu }{\hat{\nu }}}_{\mathrm{Thermal}}\;=\;F\left(T_{M},T_{R}\right)\;=\;-\frac{\Delta \nu }{\hat{\nu }}\;-\;K_{\epsilon }\left(\frac{T_{M}\;+\;T_{R}}{2}\right)\cdot \epsilon_{\mathrm{Al}6060}\left(T_{M},T_{R}\right)$$

**Figure 8:**
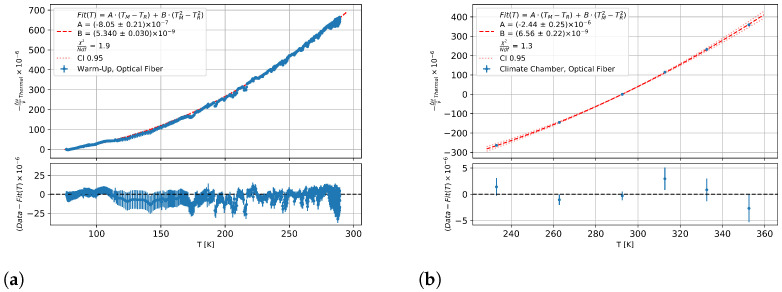
Thermal fibre signal from warm-up measurements of the optical fibre (**a**) for 77 K–290 K and (**b**) for 233–353 K against the measurement temperature with a fit of Equation (10).

The diagram in Figure 8a shows the heat signal of the germanium fibre as a function of the measurement temperature. The Equation (10) was used to fit the warm-up data. Figure 8b, on the other hand, shows the fit of the climate chamber data to another reference data set with a different $T_{R}$ to minimise the noise.

The parameters from the two adjustments are given in Table 5. The systematic uncertainty of the adjustment was determined by a joint random variation of strain, temperature, and strain sensitivity within their systematic uncertainty. An additional systematic uncertainty was related to the choice of reference temperature, which is denoted as $T_{R}$ in Equation (14). Checking the fit parameters for a $T_{R}$ dependence also gives an indication of whether the assumed theory is correct, as it does not allow for such a dependence. The results showed no significant $T_{R}$ dependence, and the scattering of the temperature sensitivity parameters for both temperature ranges was smaller than the statistical uncertainty from the adjustments in Figure 8, which were thus considered as a systematic uncertainty that was included into the overall uncertainty.

**Figure 9:**
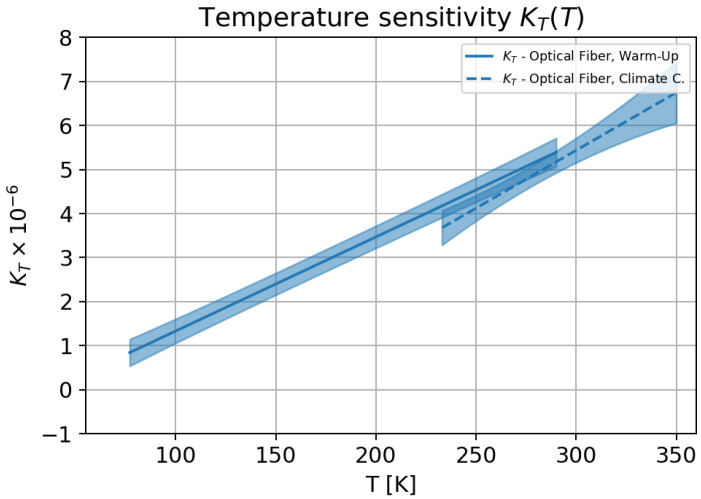
Temperatur sensitivity for the optical single-mode fibre in the temperature range 77 K–353 K.

The temperature sensitivity determined here was dependent on the strain sensitivity, which was also true for all other temperature sensitivities reported in the literature. A comparison was, therefore, difficult, since, in the literature, a constant strain sensitivity has been assumed for the determination of the temperature sensitivity. Figure 9 shows the measured temperature dependence.

The final calibrated fibre parameters for the strain and temperature coefficients with the overall uncertainty are summarised in Table 5.

## 4. Calibration Test

An independent test was carried out with a cylinder-shaped aluminium sample. It had the dimensions of 50 mm in height, 5 mm in thickness, and a diameter of 110 mm as a scaled miniature demonstrator for the future AMS-100 coil. This circular structure was chosen to show that the determined parameters were approximately independent of the chosen shape of the test object.

**Figure 10:**
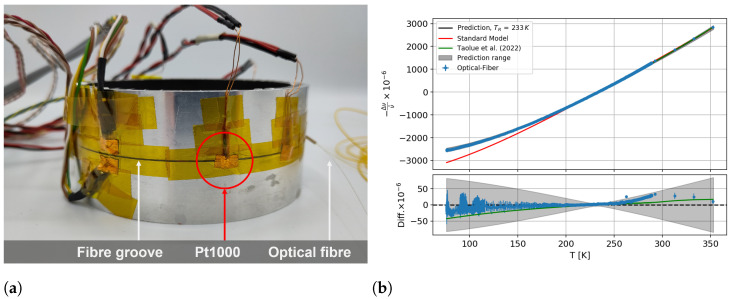
(**a**) Aluminum-ring-shaped test sample with integrated optical fibre and (**b**) measured and predicted spectral shift from a ring-shaped aluminum test sample with a reference at 233K. The standard model is shown in red, and the non-linear model from this work is shown in black with a grey prediction range. A prediction based on the parameters from Taolue et al., 2022 [19] has been adapted in green.

The optical fibre was glued into a groove with dimensions of 0.8 mm in width and 0.5 mm in depth at half the height of the cylinder. The fibre was fed in and out of the ring through 10 mm long PEEK tubes with an offset to each other. Six Pt1000 sensors were attached to the structure, and the same warm-up and climate chamber measurements were performed as were performed for the temperature sensitivity calibration. For the calibration test, a reference measurement between 77  K and 300  K was used at 233 K to show a possible reference temperature bias. An average of 20 mm with the IQR filter was used at the position of the second Pt1000 sensor, as shown in Figure 10a. By using the measured temperature and the calculated strain data, a prediction was made with Equation (12).

It can be seen in Figure 10b that the calculated prediction agreed with the measured spectral shift within the uncertainties, and the deviation was below 50 μm/m. A systematic deviation from 250 K upwards can be seen in the difference plot, which was not significant. The signal prediction based on the parameters of Taolue et al. (2022) agrees within the uncertainty with the prediction determined with the parameters and the model inserted here. One can also see that the standard model (Equation (1)) did not describe the measured optical data at temperatures below 200 K.

## 5. Discussion

As shown in this study, the investigation of the temperature and strain behaviour of fibre optic sensors in the range of 77 K to 353 K is crucial for the accurate monitoring of critical changes in structures. The results show that the fibre-specific strain coefficient was temperature-dependent and decreased at lower temperatures. In addition, the temperature coefficient exhibited a linear dependence on temperature, which must be taken into account for accurate temperature measurements. With the additional simulations carried out, it was shown that the strain applied to the aluminium sample corresponded to the fibre strain, and, thus, a calibration by the cryogenic tensile test setup was possible. The use of the custom-built cryogenic tensile test setup enabled accurate measurements of the OFDR spectral shift at different temperatures for applied external strain that were verified by strain gauge measurements. In addition, a test over the entire temperature range showed that the parameters determined here described the fibre optic data as well as the actual fibre parameters from Taolue et al., without requiring additional fibre parameters such as the Young’s modulus for their determination. Of particular importance was a detailed calibration of the strain gauges used for the relevant temperature range, as the manufacturer only specified this calibration curve for a single material with a constant alpha, so it was repeated here for different materials with a precisely known CTE temperature dependency. With these calibrated measurements, a non-linear model was parameterised to describe the temperature dependency of the OFDR spectral shift for fibres that were mechanically bonded into a carrier substrate. The need for this additional calibration resulted in an overall time-consuming characterisation, which limits the proposed method and can be improved in further studies. Moreover, the proposed method was tested for standard telecommunication single-mode fibres and provided a high resolution over a long range. It is, therefore, unclear whether this method works for other fibre types. A change in the fibre type could result in limitations such as a decrease in the measurement range due to high internal losses or lower resolution. There is also uncertainty as to whether the presented method can also work with polarisation maintaining, hollow core fibres, or multi-mode fibres, which should be investigated further in upcoming studies. The presented model with the SM9/125 fibre could be used to measure the strain of a support structure with integrated fibres and enabled the decoupling of the strain signal from the signal caused by the CTE of the material, which could thus be measured independently of the fibre CTE. This study confirms that fibre optic sensors could accurately determine the strain or temperature of an aluminium structure over the entire temperature range considered. This shows that optical fibres are a suitable solution for monitoring critical structural changes in space, such as on an AMS-100 magnetic spectrometer.

Furthermore, the measurements underline the advantages of using fibre optic sensors in extreme environmental conditions such as space. The proposed method can be used, for example, for detecting a quench in a high-temperature super-conducting coil such as in the AMS-100. It can be triggered by external mechanical or thermal influences, among other things. More likely, however, is a temperature rise caused by current not flowing through the superconducting layer for unknown reasons. If the structure can expand locally due to the thermomechanical stress, the mechanical signal becomes dominant due to the high CTE of aluminium. This signal correlation can be investigated with the proposed sensor fibres. These fibre optic sensors offer high accuracy and immunity to electromagnetic interference. Due to their low weight and compact design, they can be easily integrated into structures with little installation space. With the precise calibration of the fibre parameters, parallel measurement of the temperature and strain is also possible for known materials, even over long distances.

## 6. Conclusions

This paper shows a successful method to determine the fibre-specific coefficients for OFDR measurements to calculate strain and temperature from 77 to 353 K. A detailed calibration was performed using additional strain gauges and thermal resistors. Moreover, a nonlinear model was described to decouple strain and temperature with these calculated parameters for integrated sensor fibres in known materials. The calibration parameters could be tested independently with the linear model for a ring-shaped sample. The standard model showed a deviation from the measured parameters for colder temperatures, while the prediction with the non-linear model agreed with the measured spectral shift. This proves the calibration parameters. Overall, the results of this study provide important foundations for the development of distributed fibre optic sensor systems for space applications and demonstrate the potential of fibre optic sensor technology in extreme environments. Further research in this area should focus on improving the accuracy and precision of fibre optic sensors and exploring new applications and uses for fibre optic sensors in space exploration and other extreme environments.

## Figures and Tables

**Table 1:** Mechanical properties of the simulated materials.

Material	E-Modulus [GPa]	Poisson’s Ratio [-]
Aluminium 6060	70	0.33
Epoxy adhesive EP-50-3150	2.1	0.4
Polyimide	3.76	0.34
Fused silica	72.5	0.17

**Table 2:** Fitting parameters for temperature-dependent strain curve (see Equation (13)).

Material	$B_{0}\cdot 10^{-3}$ [K]	$B_{1}\cdot 10^{-6}$ [1/K]	$B_{2}\cdot 10^{-7}$ [$1/K^{2}$]	$B_{3}\cdot 10^{-10}$ [$1/K^{3}$]	$B_{4}\cdot 10^{-13}$ [$1/K^{4}$]
Aluminum 6060	−4.0427	−7.7712	1.4388	−3.2423	2.8743
Epoxy EP-50-3150	−11.2	29.6	−0.813	3.86	0.0
Polyimide	−8.9284	−7.379	2.7922	−6.6873	5.212

**Table 3:** Simulated strain for the aluminium sample and the optical fibre under different temperatures.

Temperature	Strain $\epsilon_{z}$ Aluminium $\cdot 10^{-6}$	Strain $\epsilon_{z}$ Fibre $\cdot 10^{-6}$
77 K	−4655.55	−4687.98
100 K	−4211.29	−4241.45
200 K	−2540.49	−2571.81
300 K	167.44	170.72

**Table 4:** Measured values for strain sensitivities for the optical fibre with the calculated statistical (stat) and systematic (sys) errors.

Temperature [K]	Strain Sensitivity
300	$8.2900\cdot 10^{-1}\;\pm \;0.82\cdot 10^{-3}$ (stat) $\;\pm \;5.0\cdot 10^{-3}$ (sys)
77	$7.917\cdot 10^{-1}\;\pm \;1.0\cdot 10^{-3}$ (stat) $\pm \;4.7\cdot 10^{-3}$ (sys)

**Table 5:** Measured parameters for strain and temperature for the optical fibre with the overall uncertainty for the temperature range from 77 K–353 K.

Parameter	Sensitivity Parameters	Uncertainty
$K_{\epsilon }$(T) (77–353 K)	$1.72\cdot 10^{-4}\;+\;0.7775\;\cdot \;T$	0.0052
$K_{T}$(T) (77–290 K)	$-0.805\cdot 10^{-6}\;+\;5.340\cdot 10^{-9}\cdot T$ [1/K]	0.26 [1/K]
$K_{T}$(T) (233–353 K)	$-2.44\cdot 10^{-6}\;+\;6.56\cdot 10^{-9}\cdot T$ [1/K]	0.36 [1/K]

## Data Availability

The data presented in this study are available on request from the corresponding author.
